# Genetic diversity of *Salmonella enterica* subsp. *enterica* serovar Enteritidis in the Siberia and Far East of Russia based on plasmid profiles

**DOI:** 10.3934/microbiol.2020007

**Published:** 2020-05-08

**Authors:** Alexey V. Rakov, Natalya A. Kuznetsova, Anatoly A. Yakovlev

**Affiliations:** Laboratory of Molecular Epidemiology and Ecology of Pathogenic Bacteria, Somov Institute of Epidemiology and Microbiology, Vladivostok, Russia

**Keywords:** *Salmonella*, Russia, genetic diversity, molecular epidemiology, plasmids

## Abstract

For the first time, in the literature review we presents the molecular genetic structure of *Salmonella* Enteritidis populations in Russia, and particularly, in Siberia and the Far East of the country. Pathogen population in Russia has been compared with *Salmonella* populations circulating in different countries of the world. It has been shown that the microbial population is heterogeneous, but it is possible to identify the dominant and main genotypes, which determine up to 90% of the total population morbidity. The data were obtained as a result of a 30-year monitoring (1988 to 2018) by studying the microbial plasmid profiles. It was shown that the same *S.* Enteritidis clones circulate throughout Russia, however, their significance in the population morbidity may vary depending on geographic and temporal characteristics. Population is characterized by heterogeneity and relative stability of the plasmid types' structure. At the same time, the population is also specified by variability, reflected as a simple change of the annual number of constantly detected plasmid types, and the appearance of new ones that can play a significant role in the etiology of *Salmonella* infection.

## 1. Introduction

In modern microbiology, much attention is paid to studying the population heterogeneity of infectious diseases pathogens, which opens up prospects for the insight of ecological and epidemiological patterns of the epidemical situation and the development of fundamentally new methods of diagnostics, treatment and prevention.

This direction of research has created two main approaches to the analysis of pathogen populations: microbiological and molecular genetics. The microbiological methods of typing existing in the modern period characterize the heterogeneity of pathogenic species by their phenotypes, and the significance of these methods has not decreased to the present. Molecular genetic typing methods reveal the heterogeneity of microbial species according to the molecular characteristics of their chromosomal and extrachromosomal parts of the pathogen's genome.

*Salmonella enterica* occupies a leading place in the etiology of human bacterial intestinal infections. The main subspecies of this species is *enterica*, which in turn is divided into more than 2,600 serovars [1]. *Salmonella enterica* subsp. *enterica* is highly adapted to warm-blooded higher vertebrates. However, according to the degree of their adaptation to the host organism, this species can be divided into two large groups: specific (host-adapted) and non-specific (host-unadapted) [2]. The first group includes several serovars (*S.* Typhi, *S.* Paratyphi, *S.* Sendai, *S.* Abortusequi, *S.* Gallinarum, *S.* Pullorum, *S.* Typhisuis, *S.* Abortusovis, *S.* Dublin and *S.* Choleraesuis), which are usually cause severe systemic diseases. All other serovars belong to the second group, and first of all, *S.* Enteritidis and *S.* Typhimurium, which in the modern period occupy leading positions in the etiology of the disease in humans. Their epidemiological significance may vary depending on the year and the area of distribution [3],[4]. However, in most cases, *S.* Enteritidis is the main serovar in the etiology of the disease in humans in many countries of the world [5], including Russia [6].

In recent years, it became known that *Salmonella* serovars specific to a particular host are genetically less diverse, i.e. characterized by fewer alleles and frequent absence (pseudogenization) of genes than non-specific serovars [7]. Since *Salmonella* is an intracellular facultative pathogen, it is possible that some chromosomal genes may be lost, the function of which can be performed by the host genes to which this serovar is adapted [8]–[11].

Besides chromosomes, it is known that plasmids and their presence also determine the genetic diversity of microorganisms [12]. In *Salmonella enterica* subsp. *enterica*, plasmids are found in the majority of serovars. For serovar *S.* Enteritidis, plasmids were detected in 98% of isolates [13], and in 94% of strains isolated from patients with *Salmonella* infection, the serovar-specific virulence plasmid pSEV with a size of 59,372 base pairs (bp) has been found [14]. It has been shown that this plasmid is fairly conservative, therefore its use in estimating genetic diversity is limited [14]. However, in addition to the virulence plasmid, *S.* Enteritidis strains have a lot of other plasmids of various sizes, the presence or absence of which determines the plasmid type of the pathogen. It makes possible to use them as an epidemiological markers for the pathogen monitoring. The monitoring system allows one or several complementary molecular genetic methods to evaluate the microbial population structure and the contribution of individual genotypes to the population morbidity in a given territory, which allows for more targeted preventive control measures [6].

The theoretical basis of the monitoring system is the development of a clonal concept of the microbial population structure, according to which the structural unit of the population is a clone [15]. From the evolutionary genetic point of view, a clone is defined as a group of genetically identical or closely related cells that are identical by origin from a common ancestral cell in the absence of chromosomal recombination [16]. In accordance with this definition, natural populations are a combination of, more or less, independent cell lines with known genetic markers, the analysis of which allows one to track the spread of individual genotypes of the microbe.

## 2. Characteristics of the molecular genetic diversity of *Salmonella* in various regions of the world

### 2.1. Research methods and history of studying the molecular genetic diversity of *Salmonella*

We think that the history of the study of the *Salmonella* molecular diversity may be counted from the early 1970s. During these years, rather simple methods for the isolation of plasmid DNAs were developed, which subsequently allowed them to be electrophoretically separated in agarose gel [17]. It allowed discovering that large-sized plasmids found in many strains of the same serovar were associated with virulent properties [18],[19]. In addition to the fact that plasmid profile analysis was often used to interpret outbreaks of infection and for monitoring of the pathogen, the population structure for each of the individual *Salmonella* serovars was for the first time identified. The results of plasmid profiling showed that plasmids are present in 96% of the isolates of all *Salmonella* serovars and, therefore, are able to be differentiated by this method [20],[21]. In particular, by studying of 278 *S.* Typhimurium strains, isolated in 1973–1981 in the New York state, the authors were able to differentiate four main plasmid types and select the most significant ones (dominating plasmid type of 7.5:6:3 kb) in terms of epidemiology [22]. Thus, it was always possible to isolate the dominant plasmid type(s) and types that play a secondary role in the epidemiology of infection. To overcome one of the disadvantages of plasmid profile analysis, the similarity of different plasmids by weight, the method of restriction endonuclease analysis of plasmids (REAP typing), allowing one to prove the similarity or difference of plasmids that are close by molecular weight, was introduced [23].

In the mid-1980s–early 1990s a much useful information about the genetic structure of *Salmonella* populations was obtained using restriction enzyme analysis of chromosomal DNA and specifically its variant, Southern blot analysis, in which probes are used as a marker [24]. Another method, ribotyping is the most common variant of the Southern blot analysis [25], in which the nucleotide sequences of the 16S and 23S rRNA genes used as probes [26].

Since the early 1990s for *Salmonella* differentiation, PCR typing methods were developed and widely used. Molecular markers used for typing include individual genes, IS*200* elements [27] and repeated nucleotide sequences that are scattered throughout the genome. The latter include in ERIC-PCR (Rep-PCR) and RAPD-PCR methods. In ERIC-PCR (enterobacterial repetitive intergenic consensus, Rep-PCR), repeated nucleotide sequences from genomic DNA are amplified [28]. Two main types of repeats are used for typing: repetitive extragenic palindromic (REP) elements and intragenic constant sequences. Both variants have a good differentiating ability at the strain level; therefore ERIC-PCR is a widely used method of DNA typing. PCR using random primers (RAPD - random amplified polymorphic DNA) is based on the use of short random oligonucleotide primers with a length of 9–10 bp, which hybridize with the DNA target at a low annealing temperature. In this variant of PCR typing, short primers and a low annealing temperature are used to initiate amplification of nucleotide sequences in different regions of the genome [29].

Until 2010 pulse-field gel electrophoresis of chromosomal DNA (PFGE) was the ‘gold standard’ for *Salmonella* typing [30]. It gained its greatest popularity in the 1990s, when it was introduced into the PulseNet monitoring system at the CDC in the United States [31]. The results of studies using from one to three restriction endonucleases showed its high resolution and advantage over most other phenotypic and genotypic methods of intraspecific typing [32].

In the early 2000s sequencing of the first complete *Salmonella* genomes has made it possible to identify loci where microsatellite repeats are concentrated, which in turn led to a new method known as multilocus variable number tandem repeat analysis (MLVA) [33]. The method is based on the difference in the number of repeats in DNA belonging to different clonal lines, using capillary electrophoresis or Sanger sequencing [34]. Currently, this method, along with whole genome sequencing (WGS), is complementary to PFGE in the monitoring system for *Salmonella* in the CDC [35].

At the same time, the technology of multilocus sequencing typing (MLST), based on Sanger sequencing of a number of household gene fragments, was developed [36]. This technique has ambiguously proven itself when typing *Salmonella*, showing that different serovars are not sufficiently diverse compared to the population structure detected by the PFGE [37]. Due to the high portability of this technique, a publicly accessible worldwide online database of sequence types has appeared, allowing phylogenetic analysis of a large number of isolates, combining related sequence types into clonal complexes [38].

Due to the availability of next generation sequencing (NGS) methods, whole genome sequencing (WGS) is currently the most advanced, and at the same time, the most expensive method [39]. The online database EnteroBase, which also included a legacy database of sequence types, allows for the analysis of any level of complexity [40] on more than 250,000 *Salmonella* genomes. However, the bottleneck of the building of such databases is the lack of metadata, the exhaustiveness of which completely depends on researchers who deposits the results of their research to the database.

### 2.2. The results of a study of *S.* Enteritidis population using plasmid profile analysis

Among all the above-described methods, plasmid analysis is the first, the simplest and the most used one for all years since molecular genetic monitoring of *Salmonella* was introduced, and *S.* Enteritidis is the most common serovar among all *Salmonella enterica* subsp. *enterica*. Taking this into account, in this review we present the study results of the *S.* Enteritidis population using plasmid profile analysis.

The use of the molecular genetic methods of intraspecific typing of *S.* Enteritidis presented above allowed us to obtain new information, first of all, about the microbe population structure in various regions of the world. One of the first ideas about the heterogeneity of a microbe population was obtained by plasmid profile analysis of 219 *S.* Enteritidis strains isolated in 1988 from patients in Spain [41]. The studied strains were represented by 15 plasmid types of the microbe, among which two plasmid types dominated, containing different sets of plasmids, which accounted for 82.6% of the strains. The most frequently detected was a plasmid type carrying a single plasmid of 59 kb, which found in 128 strains (58.4%). The second most frequent were the strains containing plasmids 59 kb and 3.0 kb, which accounted for 53 strains (24.2%). The presented heterogeneity of the *S.* Enteritidis population persisted in 1990, when strains containing the 59 kb plasmid, which found in 44.5%, continued to dominate among the 72 strains of the microbe isolated from the patients [41].

Similar to the results obtained in Spain on the plasmid characterization of the *S.* Enteritidis population structure were the results obtained in USA and Czech Republic. In a study of 154 *S.* Enteritidis strains isolated in the United States from humans, animals, and food, 16 plasmid types of the microbe were identified [21]. Among them, two plasmid types dominated (59 kb and 59:4.7 kb), combining 76% of the studied isolates. Among the 663 strains of *S.* Enteritidis studied in the Czech Republic, represented by 15 plasmid types, two plasmid types also dominated (59 kb and 59:5.2 kb) [42],[43].

Similar results on the heterogeneity of the *S.* Enteritidis population were obtained by using combination of methods for intraspecific genotyping, including plasmid profile analysis, PFGE and PCR typing. A study of the *S.* Enteritidis strains collections isolated from various sources in Poland and UK showed that the strains of the phage type PT4, most often detected in patients, may be genetically identical or their heterogeneity can be largely determined. For example, in the study by the PFGE and plasmid profile analysis of 140 *S.* Enteritidis strains isolated in Poland [44], four microbe genotypes were identified. However, all the *S.* Enteritidis strains of the phage type PT4 were identical by the plasmid profile and by the results of PFGE typing. In England [45], where the microbe of the phage type PT4 also plays a leading role in the etiology of the infection, 30 strains of the phage type PT4 were found to have 12 genotypes, but 22 strains (73%) belonged to the one leading genotype. It must be emphasized that this limited heterogeneity of the microbial population is associated not only with the phage type PT4. *S.* Enteritidis PT11 and PT9a strains isolated in Germany in 1994–1998 were also closely related by using plasmid profile analysis and PFGE [46].

A comparable level of heterogeneity of *S.* Enteritidis strains was found to be characteristic of isolates from Southeast Asia (Hong Kong, Taiwan, Japan). A study using plasmid profile analysis, ribotyping and RAPD-PCR of 264 *S.* Enteritidis strains isolated in 1986–1996 from patients in Hong Kong [47] showed that 219 isolates (83%) were of the same 59 kb plasmid type. Generally, 90% of the isolates were closely related according to the results of ribotyping, which indicated circulation of related *S.* Enteritidis strains in Hong Kong during the period of study. In Taiwan [48], a plasmid profiling of 71 *S.* Enteritidis strains revealed five plasmid types. Nevertheless, the dominant plasmid type of 59 kb was detected in 80% of the strains.

It is needed to emphasize here that in the references given above the size of the 59 kb virulence plasmid can vary from 50 to 60 kb due to the lack of consistency of exact plasmid size in the 1980s.

Thus, the presented results showed that the *S.* Enteritidis population in different regions of the world is heterogeneous. At the same time, usually the degree of *S.* Enteritidis heterogeneity was limited due to the presence of one or two dominant plasmid types in the studied collections of strains, which account for the majority of the studied cultures. The most common plasmid type was *S.* Enteritidis, containing one single virulence plasmid of 59 kb. Overall, the remaining genotypes of the pathogen in collections were represented by single isolates.

At the same time, as can be seen from the presented literature, only individual collections of *S.* Enteritidis strains were studied. They were often collected from different periods of time, and, usually they were very few in number. Therefore, the studies did not analyze the role of individual genotypes in the epidemiology of infection. The high cost and labor intensity of most of the above-described methods of intraspecific genotyping of *Salmonella* exclude the possibility of their use in the long-term monitoring of a large amount of *Salmonella* isolates [49].

## 3. Molecular genetic diversity of *Salmonella* in Russia

Unfortunately, due to the fact that Russia is still not a member of PulseNet International (PulseNet Europe) surveillance system, data on the genetic diversity of *Salmonella* serovars and other foodborne bacterial pathogens (*Escherichia coli, Shigella, Listeria, Campylobacter, Vibrio cholerae, Vibrio parahaemolyticus, Yersinia pestis*) in Russia are practically missing. Those few studies carried out in Russia with the use of PFGE using other (non-CDC) protocols and other methods are mostly limited to the study of strains isolated during salmonellosis outbreaks and are not presented in the available peer-reviewed journals. A systematic long-term study of the molecular genetic diversity of *Salmonella* in Russia is carried out only at the Somov Institute of Epidemiology and Microbiology (Vladivostok), which began in the 1990s with the study of *Salmonella* strains isolated in the Primorye Region. Since 2000, molecular genetic monitoring has been gradually extended to other regions of the Far East of Russia, and then of Siberia. The monitoring is based on the plasmid profile analysis, as the most affordable method for the study of a large amount of *Salmonella* isolates submitting to the laboratory from the above-specified areas (more than 1,000 strains annually). Currently, an abundant material has been accumulated on the genetic diversity of *Salmonella* circulating in Siberia and the Far East, which is shown below.

The etiological structure of *Salmonella* infection in the Primorye Region, as well as throughout the USSR, changed in circa 1987 as a result of the replacement of the dominant serovar *S.* Typhimurium to serovar *S.* Enteritidis. Since 1988, *Salmonella* infection caused by *S.* Enteritidis began to dominate in the structure of the morbidity, and its relative share reached an average of 95.5%. This led to the emergence of numerous outbreaks of disease and a six-fold increase in the *Salmonella* infection incidence in the USSR [50]. A similar process took place in other countries, but it began earlier in the 1970s [51],[52]. Plasmid profile analysis of *S.* Enteritidis strains isolated from Vladivostok, Primorye Region, from 102 patients in 1988–1991, showed that they all carried a single virulence plasmid of 59 kb and belonged to the same plasmid type [53].

Further changes in the *S.* Enteritidis population structure were identified in 1991, when, during the study of microbial strains isolated from 86 patients, a plasmid type 59 kb was detected in 80 patients, whereas the strains isolated from the remaining six patients belonged to four previously unreported (new) plasmid types (94:59 kb, 94:59:3.2 kb, 59:3.2 kb, and 73:59:45 kb) [13].

**Figure 1.**
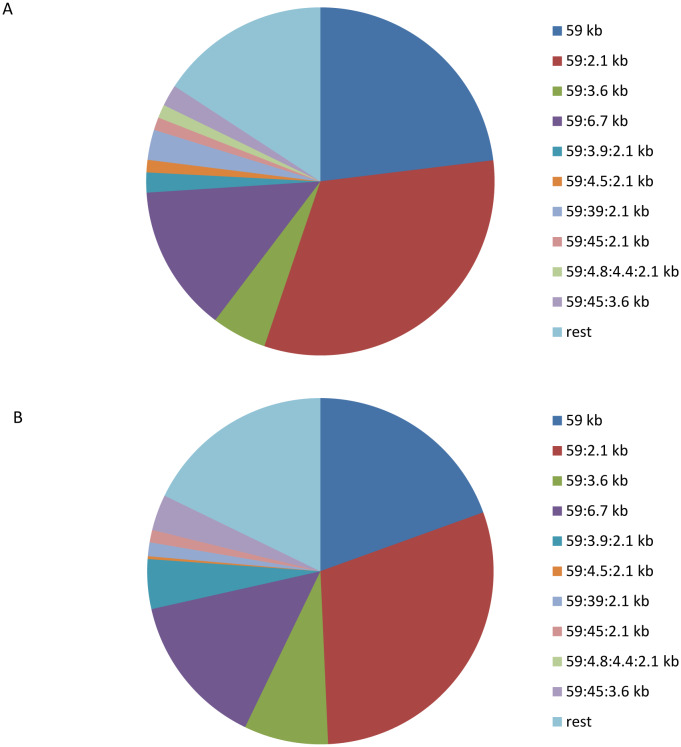
Pie chart showing the relative incidence of the *S.* Enteritidis population structure by plasmid types: (A) isolated from patients with *Salmonella* infection in Siberia and the Far East from 2000 to 2018; (B) isolated from food for the same period of time.

The presented results were the basis for a focused study of the dynamics of the salmonellosis incidence in human population in accordance with the plasmid characteristics of *S.* Enteritidis strains isolated from patients, which was done in the process of microbiological monitoring of the pathogen from 1995 to the present. The results obtained over the years of molecular genetic monitoring, indicating the heterogeneity of *Salmonella* populations in different territories, are reflected in a number of publications [3],[21],[53]–[60]. During the analyzed period 22,075 strains of *S.* Enteritidis were studied, which accounted for 70% of all strains isolated from various sources. This included strains isolated: 20,889 from humans, 1,148 from product samples, 27 from environment and 11 from synanthropic rodents. The results of the study of the etiological significance of various *S.* Enteritidis plasmid types in the period 1995–2018 are presented in Figure 1.

Plasmid analysis of *S.* Enteritidis strains revealed significant heterogeneity of the population by the plasmid profile. In total, among the strains isolated in 1995–2018 it was revealed about 500 plasmid types that were differentiated into two groups according to the frequency of occurrence. The first group included strains of 10 plasmid types, the share of which in different years of observation accounted from 86.5% to 97.1% of the isolated strains. They were designated as ‘main’ plasmid types. All other strains, which were isolated much less frequently, were assigned to the second group and designated by us as ‘rarely detected’ [54].

The strains of the main *S.* Enteritidis plasmid types differed in the frequency of isolation (Figure 1). Firstly, three types of microbe were isolated from patients, marked with plasmids 59 kb, 59:2.1 kb and 59:6.7 kb. Secondly, *S.* Enteritidis of these three types was isolated during almost the entire observation period, and they accounted from 72.4% to 90.3% of the isolated strains. These plasmid types were designated as ‘dominant’. The key feature of the dominant plasmid types of the pathogen is that they played a leading role in the etiology of salmonellosis caused by *S.* Enteritidis during all the years of surveillance in all observed territories [54].

The remaining seven plasmid types assigned to the main group (59:3.6 kb, 59:3.9:2.1 kb, 59:4.5:2.1 kb, 59:39:2.1 kb, 59:45:2.1 kb, 59:4.8:4.4:2.1 kb, 59:45:3.6 kb) were isolated much less frequently, and their etiological significance during the years of surveillance varied considerably.

As can be seen from Figure 1, there is a clear link between the plasmid types of the microbe isolated from food (Figure 1B) and isolated from patients (Figure 1A), which suggests the foodborne route of transmission.

A separate group consisted of strains of rarely detectable plasmid types of *S.* Enteritidis. Strains of this category were annually represented by a significant number of plasmid types. Their key feature was that they were not significant for the morbidity formation of the population, since in most cases they were isolated from the sporadic patients. Moreover, the annual total etiological significance of rare plasmid types throughout the entire observation period remained low and ranged from 2.6% to 13.5%.

The second plasmid type, which subsequently, along with the plasmid type 59 kb, occupied a dominant position, was 59: 2.1 kb. First identified in 1995, this plasmid type caused a sharp increase in the incidence rate, which was accompanied by a more severe disease [49], and subsequently began to occupy a leading role in the incidence rate (from 13.4% to 54.8% of the total incidence of salmonellosis depending on year) until now.

The third most important type was 59:6.7 kb, although it was isolated in insignificant quantities in 1998–2001 and 2003, as well as type 59:2.1 kb in 1995, causing a sharp increase in the incidence rate, with time it took the third place with a share of 8.22% to 31.8% of the total incidence.

As can be seen from Figure 3, differences in the *Salmonella* population structure between the regions of the southern and northern administrative territories of the Far East and Siberia were insignificant [61]. In all regions, dominance of the two main plasmid types 59 kb and 59:2.1 kb was observed. The third main plasmid type 59:6.7 kb prevailed in the Primorye Region, as well as in the Kamchatka Region and the Magadan Region, which may be associated with the supply of poultry products to these regions from the Primorye Region [57]. Most of the dominant plasmid types were isolated in all territories of Siberia and the Far East.

**Figure 2.**
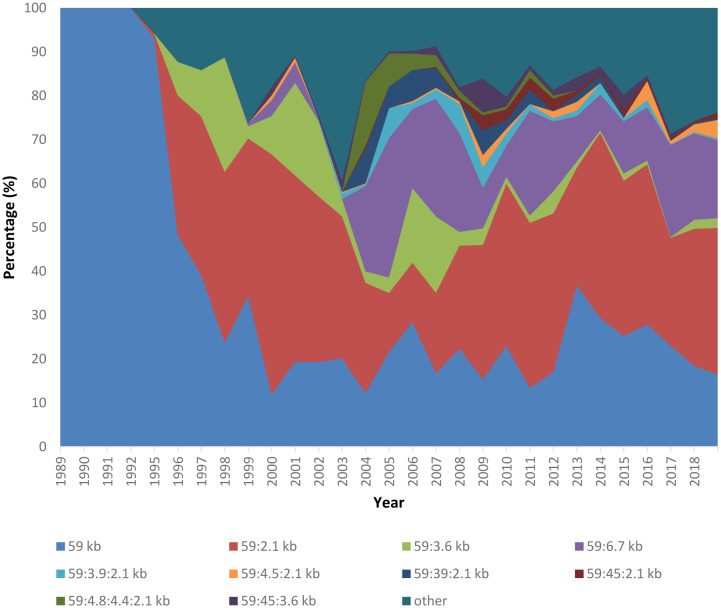
The dynamics of *S.* Enteritidis plasmid types isolation from patients in Siberia and the Far East of Russia from 1995 to 2018.

It should be noted that new plasmid types were detected in different years, and their share in the total amount of *S.* Enteritidis isolates from patients varied significantly depending on the year (Figure 2).

**Figure 3.**
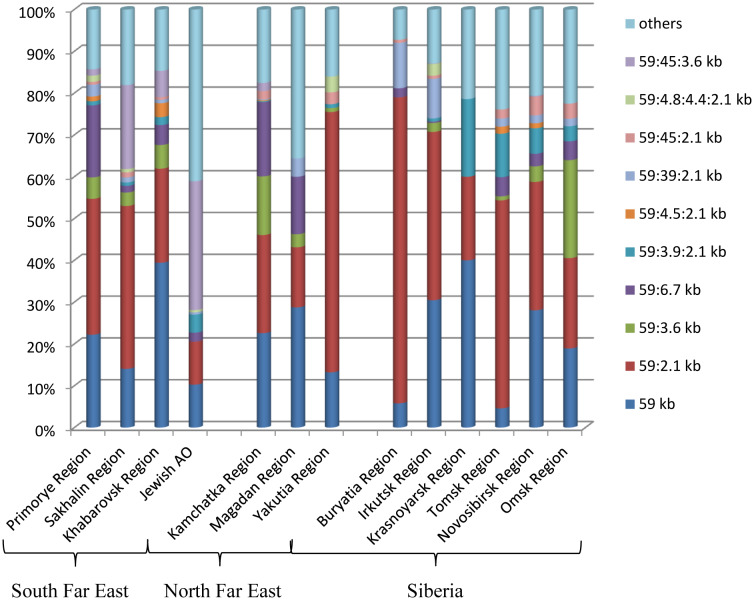
Relative share of dominant plasmid types in the etiology of *S.* Enteritidis infection in the human population of the southern and northern territories of the Far East and Siberia (of total percentage of strains isolated from 1995 to 2018).

In this regard, it is important to emphasize that our studies of *S.* Enteritidis strains isolated in the European part of Russia (Murmansk, Izhevsk) showed that their plasmid profile is the same as that of the populations circulating in Siberia and the Far East [62].

## 4. Conclusion

Thus, characterization of the *S.* Enteritidis population in the period 1995–2018 is highlighted its main properties. First of all, the population is characterized by heterogeneity and relative stability of the structure, which is determined by the presence of the main and rarely detected plasmid types of pathogen. From the epidemiological point of view, the heterogeneity of the population is evidenced from the different epidemic significance of plasmid types with the leading role of the main ones, which are dominant in the etiology of *Salmonella* infection. When comparing plasmids and plasmid types, it was found that the plasmid type, containing single 59 kb virulence plasmid, is dominant in most countries of the world, including Russia. A 2.1 kb plasmid pJ was described in the composition of the plasmid types of the pathogen isolated in the Czech Republic [42] and the USA [56], which led to the conclusion that this plasmid has transcontinental distribution [56].

Moreover, the population is characterized by variability, evidenced from a simple change of the annual number of constantly detected plasmid types, and the emergence of new ones that can play a significant role in the etiology of *Salmonella* infection. Presumably, changes in molecular genetic heterogeneity are a continuous process that allows microorganisms to adapt to changing environmental conditions, which, in turn, is reflected in the genome plasticity [63]. At the same time, this phenomenon may be due to inter-and intraspecific interaction of *Salmonella*, as a result of their self-regulation in the biogeocenosis [64],[65].

Thus, the same *S.* Enteritidis clone circulate throughout Russia, however, their significance in the morbidity of the population may vary depending on temporal and, to a lesser extent, on geographical features.

## References

[1] Ryan MP, O'Dwyer J, Adley CC (2017). Evaluation of the complex nomenclature of the clinically and veterinary significant pathogen *Salmonella*. BioMed Res Int.

[2] Lamas A, Miranda JM, Regal P (2018). A comprehensive review of non-*enterica* subspecies of *Salmonella enterica*. Microbiol Res.

[3] Shubin FN, Rakov AV, Kuznetsova NA (2015). Zoonotic salmonellosis in the Far East: main aspects of a problem. Far-Eastern J Infect Pathol.

[4] EFSA and ECDC (European Food Safety Authority and European Centre for Disease Prevention and Control) (2018). The European Union summary report on trends and sources of zoonoses, zoonotic agents and food-borne outbreaks in 2017. EFSA J.

[5] Hendriksen RS, Vieira AR, Karlsmose S (2011). Global monitoring of *Salmonella* serovar distribution from the world health organization global foodborne infections network country data bank: results of quality assured laboratories from 2001 to 2007. Foodborne Pathog Dis.

[6] Shubin FN (2015). Zoonotic salmonellosis in Russia: main aspects of a problem. Epidemiol Vaccinal Prev.

[7] Rakov AV, Mastriani E, Liu SL (2019). Association of *Salmonella* virulence factor alleles with intestinal and invasive serovars. BMC Genomics.

[8] Dagan T, Blekhman R, Graur D (2006). The ‘domino theory’ of gene death: gradual and mass gene extinction events in three lineages of obligate symbiotic bacterial pathogens. Mol Biol Evol.

[9] Nuccio SP, Bäumler AJ (2014). Comparative analysis of *Salmonella* genomes identifies a metabolic network for escalating growth in the inflamed gut. mBio.

[10] Valenzuela LM, Hidalgo AA, Rodríguez L (2015). Pseudogenization of *sopA* and *sopE2* is functionally linked and contributes to virulence of *Salmonella enterica* serovar Typhi. Infect Genet Evol.

[11] Ortega AP, Villagra NA, Urrutia IM (2016). Lose to win: *marT* pseudogenization in *Salmonella enterica* serovar Typhi contributed to the surV-dependent survival to H_2_O_2_, and inside human macrophage-like cells. Infect Genet Evol.

[12] Call DR, Kang MS, Daniels J (2006). Assessing genetic diversity in plasmids from *Escherichia coli* and *Salmonella enterica* using a mixed-plasmid microarray. J Appl Microbiol.

[13] Shubin FN, Kovalchuk NI, Kuznetsova NA (2002). Microbiological monitoring for *Salmonella enteritidis* in Primorye Region. Phenotypical and plasmid characterization of the pathogen. Epidemiol Infect Dis.

[14] Rakov AV, Shubin FN (2019). Comparative genomics analysis of *Salmonella enterica* subsp. *enterica* serotype Enteritidis virulence plasmid. Russ J Genet.

[15] Orskov F, Orskov I (1983). Summary of a workshop on the clone concept in the epidemiology, taxonomy and evolution of the *Enterobacteriaceae* and other bacteria. J Infect Dis.

[16] Selander RK, Caugant DA, Whittman TS (1987). Genetic structure and variation in natural populations of *Escherichia coli*. Cell Mol Biol.

[17] Kado CI, Liu ST (1981). Rapid procedure for detection and isolation of large and small plasmids. J Bacteriol.

[18] Jones GW, Rabert DK, Svinarich DM (1982). Association of adhesive, invasive, and virulent phenotypes of *Salmonella* Typhimurium with autonomous 60-megadalton plasmids. Infect Immun.

[19] Nakamura M, Sato S, Ohya T (1985). Possible relationship of a 36-megadalton *Salmonella* Enteritidis plasmid to virulence in mice. Infect Immun.

[20] Rivera MJ, Rivera N, Castillo J (1991). Molecular and epidemiological study of *Salmonella* clinical isolates. J Clin Microbiol.

[21] Rodrigue DC, Cameron DN, Puhr ND (1992). Comparison of plasmid profiles, phage types, and antimicrobial resistance patterns of *Salmonella* Enteritidis isolates in the United States. J Clin Microbiol.

[22] Holmberg SD, Wachsmuth IK, Hickman-Brenner FW (1984). Comparison of plasmid profile analysis, phage typing, and antimicrobial susceptibility testing in characterizing *Salmonella* Typhimurium isolates from outbreaks. J Clin Microbiol.

[23] Farrar WE (1983). Molecular analysis of plasmids in epidemiologic investigation. J Infect Dis.

[24] Southern EM (1975). Detection of specific sequences among DNA fragments separated by gel electrophoresis. J Mol Biol.

[25] Olsen JE, Skov MN, Threlfall EJ (1994). Clonal lines of *Salmonella enterica* serotype Enteritidis documented by IS200-, ribo-, pulsed-field gel electrophoresis and RFLP typing. J Med Microbiol.

[26] Guerra B, Landeras E, Gonzalez-Hevia MA (1997). A three-way ribotyping scheme for *Salmonella* serotype Typhimurium and its usefulness for phylogenetic and epidemiological purposes. J Med Microbiol.

[27] Stanley J, Baquar N, Threlfall EJ (1993). Genotypes and phylogenic relationships of *Salmonella* Typhimurium are defined by molecular fingerprinting of IS*200* and 16S rrn loci. J Gen Microbiol.

[28] Hulton CS, Higgins CF, Sharp PM (1991). ERIC sequences: a novel family of repetitive elements in the genomes of *Escherichia coli*, *Salmonella* Typhimurium and other enterobacteria. Mol Microbiol.

[29] Hilton AC, Banks JG, Penn CW (1996). Random application of polymorphic DNA (RAPD) of *Salmonella*: strain differentiation and characterization of amplified sequences. J Appl Bacteriol.

[30] Schwartz DC, Cantor CR (1984). Separation of yeast chromosome-sized DNAs by pulsed gel electrophoresis. Cell.

[31] Swaminathan B, Barrett TJ, Hunter SB (2001). PulseNet: the molecular subtyping network for foodborne bacterial disease surveillance, United States. Emerg Infect Dis.

[32] Ridley AM, Threlfall EJ, Rowe B (1998). Genotypic characterization of *Salmonella* Enteritidis phage types by plasmid analysis, ribotyping, and pulsed-field gel electrophoresis. J Clin Microbiol.

[33] Ramisse V, Houssu P, Hernandez E (2004). Variable number of tandem repeats in *Salmonella enterica* subsp. *enterica* for typing purposes. J Clin Microbiol.

[34] Boxrud D, Pederson-Gulrud K, Wotton J (2007). Comparison of multiple-locus variable-number tandem repeat analysis, pulsed-field gel electrophoresis, and phage typing for subtype analysis of *Salmonella enterica* serotype Enteritidis. J Clin Microbiol.

[35] Liu Y, Shi X, Li Y (2016). The evaluation and application of multilocus variable number tandem repeat analysis (MLVA) for the molecular epidemiological study of *Salmonella enterica* subsp. *enterica* serovar Enteritidis infection. Ann Clin Microbiol Antimicrob.

[36] Kotetishvili M, Stine OC, Kreger A (2002). Multilocus sequence typing for characterization of clinical and environmental *Salmonella* strains. J Clin Microbiol.

[37] Achtman M, Wain J, Weill FX (2012). Multilocus sequence typing as a replacement for serotyping in *Salmonella enterica*. PLoS Pathog.

[38] Feil EJ, Li BC, Aanensen DM (2004). eBURST: inferring patterns of evolutionary descent among clusters of related bacterial genotypes from multilocus sequence typing data. J Bacteriol.

[39] Deng X, Shariat N, Driebe EM (2015). Comparative analysis of subtyping methods against a whole-genome-sequencing standard for *Salmonella enterica* serotype Enteritidis. J Clin Microbiol.

[40] Alikhan NF, Zhou Z, Sergeant MJ (2018). A genomic overview of the population structure of *Salmonella*. PLoS Genet.

[41] Rivera MJ, Rivera A, Castillo J (1993). Plasmid profile in epidemiological studies of human *Salmonella* infections. J Chemother.

[42] Rychlik I, Karpiskova R, Faldynova M (1998). Computer-assisted restriction endonuclease analysis of plasmid DNA in field strains of *Salmonella* Enteritidis. Can J Microbiol.

[43] Rychlik I, Svestkova A, Karpiskova R (2000). Subdivision of *Salmonella enterica* serovar enteritidis phage types PT14b and PT21 by plasmid profiling. Vet Microbiol.

[44] Cieslik A, Brown D, Paciorek J (2001). Phage types, plasmid profiles and chromosomal restriction profiles of *Salmonella enterica* subsp. *enterica* ser. Enteritidis (*S.* Enteritidis) isolated in Poland in 1999–2000. Med Dosw Microbiol.

[45] Desai M, Threlfall EJ, Stanley J (2001). Fluorescent amplified-fragment length polymorphism subtyping of the *Salmonella enterica* serovar Enteritidis phage type 4 clone complex. J Clin Microbiol.

[46] Nauerby B, Pedersen K, Dietz HH (2000). Comparison of Danish isolates of *Salmonella enterica* serovar enteritidis PT9a and PT11 from hedgehogs (Erinaceus europaeus) and humans by plasmid profiling and pulsed-field gel electrophoresis. J Clin Microbiol.

[47] Ling JM, Koo IC, Kam KM (1998). Antimicrobial susceptibilities and molecular epidemiology of *Salmonella enterica* serotype Enteritidis strains isolated in Hong Kong from 1986 to 1996. J Clin Microbiol.

[48] Su LH, Chiu CH, Wu TL (2002). Molecular epidemiology of *Salmonella enterica* serovar Enteritidis isolated in Taiwan. Microbiol Immunol.

[49] Shaginyan IA (2000). Role and significance of molecular methods in epidemiological analysis of nosocomial infections. Clin Microbiol Antimicrob Chemother.

[50] Khazenson LB, Poplavskaia ZhV, Kariagina EI (1996). Epidemiological data on salmonellosis due to *Salmonella* enteritidis in some areas of the Russian Federation. J Microbiol Epidemiol Immunobiol.

[51] Rodrigue DC, Tauxe RV, Rowe B (1990). International increase in *Salmonella* Enteritidis: a new pandemic?. Epidemiol Infect.

[52] Bäumler AJ, Hargis BM, Tsolis RM (2000). Tracing the origins of *Salmonella* outbreaks. Science.

[53] Rakov AV, Shubin FN, Ivanis VA (2001). Comparative characterization of infections caused by *Salmonella* enteritidis of different plasmid profiles. Epidemiol Infect Dis.

[54] Shubin FN, Rakov AV, Kuznetsova NA (2006). Structure of *Salmonella* enteritidis population in Primorye Region on the plasmid analysis data. J Microbiol Epidemiol Immunobiol.

[55] Shubin FN, Rakov AV, Kuznetsova NA (2011). Microbiological molecular genetic monitoring of enteric infection pathogens as an element of epidemiological surveillance. Bulletin SB RAMS.

[56] Rakov AV, Shubin FN, Kuznetsova NA (2013). Heterogeneity of 1.4 MDa plasmids in *Salmonella* enteritidis strains. Bulletin SB RAMS.

[57] Shubin FN, Rakov AV, Kuznetsova NA (2017). Formation of population morbidity with salmonellosis caused by *Salmonella* enteritidis in regions with incomplete supply of local poultry products. J Microbiol Epidemiol Immunobiol.

[58] Shubin FN, Kuznetsova NA, Rakov AV (2018). Specific features of molecular epidemiology of imported morbidity caused by *Salmonella* Enteritidis introduced strains with specific plasmid types. Epidemiol Infect Dis.

[59] Rakov AV, Kuznetsova NA, Solovyeva AS (2018). Cluster analysis of *Salmonella* Enteritidis isolated in the Siberia and Far East of Russia. Pacific Med J.

[60] Rakov AV, Shubin FN, Kuznetsova NA (2019). Heterogeneity of 2.3 MDa plasmids in *Salmonella* Enteritidis strains. Siberian Sci Med J.

[61] Kuznetsova NA, Solovyeva AS, Rakov AV (2018). Antibiotic resistance of *Salmonella* Enteritidis strains, circulated in territory of the Siberia and Far East, at multi-year monitoring. Health Med Ecol Sci.

[62] Rakov AV, Shubin FN, Kuznetsova NA (2016). Estimated microbiological and epidemiological of the outbreak of salmonellosis in the child care institution in Murmansk. Health Med Ecol Sci.

[63] Döpfer D, Buist W, Soyer Y (2008). Assessing genetic heterogeneity within bacterial species isolated from gastrointestinal and environmental samples: how many isolates does it take?. Appl Environ Microbiol.

[64] Timchenko NF, Rakov AV, Terentyeva NA (2019). Characteristics of the mixed bacteria of the *Enterobacteriaceae* family *Yersinia pseudotuberculosis* and *Salmonella* Enteritidis *in vitro*. Health Med Ecol Sci.

[65] Yakovlev AA, Pozdeeva ES (2018). Possible mechanisms of self-regulation of parasitic systems in the biogeocenosis. Vestnik RAMS.

